# The Protective Effect of *Boschnikia rossica* Extract on Free Radical-Induced Oxidative Damage of Biomolecules and Establishment of a Method for Determining the Content of Oleanolic Acid

**DOI:** 10.3390/foods14101658

**Published:** 2025-05-08

**Authors:** Zhuohao Zhao, Mingjie Jia, Yuning An, Yihong Bao, Yuncai Zhao

**Affiliations:** 1 College of Life Sciences, Northeast Forestry University, Harbin 150040, China; 15589897121@163.com (Z.Z.);; 2 Key Laboratory of Forest Food Resources Utilization of Heilongjiang Province, Harbin 150040, China; 3 Heilongjiang Light Industry Science Research Institute, Harbin 150010, China

**Keywords:** *Boschnikia rossica* extract, oleanolic acid, RP-HPLC, biomacromolecules, lipid peroxidation

## Abstract

Oxidative stress-induced damage to biomolecules such as proteins, lipids, and DNA is closely related to chronic diseases. Developing efficient, low-toxicity, and multi-target natural antioxidants has become an important research direction in food and medicine. This study established a detection method for oleanolic acid (OA) content in *Boschnikia rossica* extract (BRE). It systematically evaluated the in vitro antioxidant activity and protective effect of *Boschnikia rossica* extract on oxidative damage of biomolecules. Firstly, the detection method based on RP-HPLC has improved the problem of low separation efficiency and high interference in detecting OA in *Boschnikia rossica*. The optimal analysis conditions were obtained by optimizing the chromatographic conditions: the chromatographic column was Agilent TC-C_18_ (250 mm × 4.6 mm, 5 μm); the mobile phase was methanol/0.4% phosphoric acid aqueous solution (85/15, *v*/*v*), pH 2.14; the column temperature was 20 °C; the flow rate was 1.2 mL/min; and the detection wavelength was 220 nm. Under these conditions, the linear relationship of OA was good within the concentration range of 100–800 mg/L, with a recovery rate of 98.88–101.46% and RSD less than 2%. The content of OA was 0.358 mg/g. Next, the in vitro antioxidant effect of BRE was tested, and it was found that BRE had reasonable scavenging rates against ABTS, DPPH, and hydroxyl radicals, with IC_50_ values of 224.32 mg/L, 58.43 mg/L, and 432.21 mg/L, respectively. In addition, BRE had significant inhibitory effects on protein oxidative degradation, carbonylation modification, lipid oxidation, and DNA oxidative damage induced by different free radicals. Finally, BRE can be a natural alternative to synthetic antioxidants and has important application value in delaying food oxidation and developing anti-aging functional foods.

## 1. Introduction

Reactive oxygen species (ROS) are byproducts of normal metabolism in aerobic organisms, and normal organisms have the function of maintaining the physiological concentration of ROS balance. When various harmful factors stimulate the body, the balance between the oxidative and antioxidant systems is disrupted, causing the accumulation of free radicals and ultimately leading to oxidative stress [1]. Excessive free radicals can cause oxidative damage to biomolecules such as proteins, lipids, and DNA, leading to the destruction of cellular structure and the occurrence of various chronic diseases [2]. Antioxidants are crucial in clearing excess free radicals and inhibiting oxidative chain reactions. Some synthetic antioxidants, such as Butylated Hydroxyanisole (BHA), are used. Butylated Hydroxytoluene (BHT) can effectively delay food oxidative degradation, but its potential liver toxicity, carcinogenicity, and bioaccumulation risk in the food chain have sparked widespread safety controversies [3]. Therefore, developing efficient and low-toxicity natural antioxidant alternatives is urgently needed in the food industry. In the study of in vitro antioxidant activity, the free radical-induced oxidative damage model of biomolecules can more accurately reflect the actual protective effect of antioxidants in organisms by directly simulating key pathological processes such as protein carbonylation, lipid peroxidation, and DNA strand breakage caused by ROS. This type of model can not only quantify the targeted protective effects of antioxidants on specific biomolecules such as proteins, lipids, and DNA but also reveal their antioxidant mechanisms under highly controllable conditions (such as free radical scavenging, metal chelation, chain reaction blockade, etc.). Numerous studies have shown that active ingredients such as triterpenoids, flavonoids, and polyphenols found in plant extracts possess antioxidant properties and significantly inhibit key food degradation processes such as lipid peroxidation and protein carbonylation [4,5]. Compared to synthetic additives, plant-based antioxidants have shown great potential for application in functional food development, natural preservation system construction, and other fields due to their low toxicity and strong antioxidant effects [6]. Consuming natural antioxidants can reduce the degree of oxidative damage in the body and lower the risk of various diseases [7,8].

*Boschnikia rossica* Fedtsch. et Flerov. is a perennial herbaceous plant of the genus Boschniakia in the family Liliaceae. It nourishes, strengthens, and prolongs life [9,10] and is widely distributed in China, India, Japan, South Korea, and Russia. Modern research has shown that *Boschnikia rossica* is rich in bioactive compounds such as terpenes, phenylethanoid glycosides, phenols, and alkaloids [11]. Oleanolic acid (OA), as a characteristic triterpenoid component of *Boschnikia rossica*, has various physiological functions such as low cytotoxicity and anti-inflammatory [12], antioxidant [13], anti-tumor [14], and hepatoprotective activity [15]. Due to its outstanding pharmacological properties, OA is an important indicator for evaluating the quality of *Boschnikia rossica* and a current research hotspot in natural medicine development.

In the study of natural plants, the quantification of active ingredients is a major concern. Currently, the methods for determining the content of OA mainly include high-performance liquid chromatography, acid–base titration, the vanillin glacial acetic acid perchloric acid colorimetric method, etc. [16,17,18]. Among them, the most widely used are high-performance liquid chromatography and the vanillin glacial acetic acid perchloric acid colorimetric method. The vanillin glacial acetic acid perchloric acid colorimetric method has been verified to be affected by impurities, resulting in poor hydrolysis efficiency and a low recovery rate. In contrast, high-performance liquid chromatography (HPLC) can effectively separate the components in a mixture and accurately quantify the content of each component. In addition, high-performance liquid chromatography has the advantages of good reproducibility, high stability, and a high recovery rate [19]. There is little research on the determination of OA content in *Boschnikia rossica*. The existing methods for detecting OA use complex chromatographic conditions and have specific requirements for the type of chromatographic column, which leads to insufficient separation of certain compounds and affects the accuracy of quantification.

This study established a rapid and accurate method for determining OA content in *Boschnikia rossica* extract by optimizing liquid chromatography conditions. The antioxidant capacity of the *Boschnikia rossica* extract was studied through a free radical scavenging assay and a β-carotene bleaching inhibition assay, and its protective effect on protein, lipid, and DNA oxidative damage induced by free radicals was studied to provide a theoretical basis for the development of high-value-added products of *Boschnikia rossica*.

## 2. Materials and Methods

### 2.1. Materials and Reagents

*Boschnikia rossica* was purchased from Daxing’anling; it was identified by Professor Liqiang Mu of Northeast Forestry University and preserved in the specimen room of Northeast Forestry University, Heilongjiang Province, China. pBR322 DNA, bovine serum albumin, 98% OA standard, and β-carotene were purchased from Shanghai Yuanye Biotechnology Co., Ltd. (Shanghai, China). HsDNA was purchased from Beijing Solaibao Technology Co., Ltd. (Beijing, China). The MDA kit was purchased from Nanjing Jiancheng Bioengineering Institute (Nanjing, China). 2,2′-azodiisobutylamidine dihydrochloride (AAPH) and linoleic acid (LA) were purchased from Aladdin Reagent (Shanghai, China). Phosphoric acid, acetic acid, ethanol, sulfuric acid, and hydrochloric acid were all analytically pure. Methanol was chromatographically pure and purchased from Tianjin Beilian Fine Chemicals Development Co., Ltd. (Tianjin, China).

### 2.2. Preparation of BRE

After grinding the *Boschnikia rossica* into fine powder using a grinder, it was filtered through a 60-mesh sieve and stored in a dark place. An amount of 5 g of *Boschnikia rossica* powder was placed in a 500 mL conical flask, and 200 mL of 95% ethanol was added. The mixture was extracted by ultrasound at a power of 180 W for 50 min, filtered, and then the filtrate was concentrated under reduced pressure and freeze-dried before being set aside.

### 2.3. Preparation of Standard Samples of OA

Stock solution: 25 mg of standard OA was weighed and dissolved in 25 mL of methanol to prepare a stock solution of standard OA with a mass concentration of 1.00 mg/mL. It was stored at −18 °C. Working fluid: An appropriate amount of standard stock solution of OA was taken, diluted step by step with methanol, and then prepared into a series of standard solutions of OA with a mass concentration of 100, 200, 400, 600, and 800 mg/L. It was stored at −18 °C.

### 2.4. Establishment and Methodological Study of RP-HPLC Analysis Method

#### 2.4.1. Optimization of Chromatographic Separation Conditions

A reverse-phase high-performance liquid chromatography system was adopted, with a detector type of an ultraviolet detector. The chromatographic column was Agilent TC-C_18_ (250 mm × 4.6 mm, 5 μm) (Agilent Technologies, Beijing, China). In this study, the mobile phase was a methanol (solvent A)—phosphoric acid solution (solvent B). The analysis conditions of OA were investigated and optimized by changing the concentration of phosphate buffer solution (0.1%, 0.2%, 0.4%, 0.6%, and 0.8%), mobile phase ratio (80/20, 85/15, 88/12, and 90/10), flow rate (0.8 mL/min, 0.9 mL/min, 1.0 mL/min, 1.1 mL/min, and 1.2 mL/min), and column temperature (15 °C, 20 °C, 25 °C, 30 °C, and 35 °C). The chromatographic separation performance was evaluated by the separation factor (α), resolution (Rs), and symmetry factor (S). The separation factor (α) is defined as the ratio of the retention factors of two adjacent peaks, calculated as α = k_2_/k_1_ (where k_1_ and k_2_ are the retention factors of the first-eluting and second-eluting peaks, respec-tively). The resolution (Rs) measures the degree of separation between two adjacent peaks and is calculated as Rs = 2(tR_2_ − tR_1_)/(W_1_ + W_2_) (where tR_1_ and tR_2_ are the retention times of the two peaks, and W_1_ and W_2_ are the baseline peak widths of the two peaks). The symmetry factor (S) is an indicator used to quantify the symmetry of a chromatographic peak, calculated as S = B/A (where A represents the horizontal distance from 10% peak height on the leading edge to the peak vertex, and B denotes the horizontal distance from the peak vertex to 10% peak height on the trailing edge.). The cri-terion for separating OA in BRE was to achieve baseline separation between the main target peak (OA) and other impurity peaks within a reasonable analysis time by opti-mizing chromatographic conditions.The evaluation standards for separation parameters are as follows: the separation factor (α) must fall within the range of 1.1–1.5; the peak symmetry (S) should be controlled between 0.9 and 1.2; and the resolution (Rs) must be greater than 2.0.

#### 2.4.2. Methodological Testing

In methodological testing, the reliability and stability of the RP-HPLC analysis method were evaluated through multiple indicators. Standard curve drawing: The standard solution of the OA series was prepared according to the experimental method in Section 2.3. The sample was analyzed, and the chromatogram was recorded. The mass concentration of OA was used as the *x*-axis, and the corresponding peak area was used as the *y*-axis for linear regression analysis. A standard curve was plotted, and the regression equation was obtained. The detection limit (LOD) was calculated at 3 times the signal-to-noise ratio, and the quantification limit (LOQ) was calculated at 10 times the signal-to-noise ratio. Stability test: BRE samples stored for 0, 2, 4, 6, 8, 12, and 24 h were tested, and the retention time and relative standard deviation (RSD) of the peak area of OA were calculated to evaluate the intermediate precision. Repeatability test: 6 samples of *Boschnikia rossica* from the same batch were weighed, and then they were extracted through ultrasonic treatment. The average mass concentration of OA in BRE was calculated, and its relative standard deviation was determined. Precision and recovery tests: BRE was subjected to spiked recovery tests at concentrations of 50 mg/L, 100 mg/L, and 200 mg/L. The recovery rate and RSD of the measured values were calculated to verify the accuracy and precision of the method.

### 2.5. Antioxidant Activity of BRE In Vitro

#### 2.5.1. ABTS Free Radical Scavenging Test

An amount of 7 mmol/L ABTS and 2.45 mmol/L K_2_S_2_O_8_ solution were prepared. After being mixed in equal proportions and reacted in the dark at 25 °C for 16 h, they were diluted to 0.7 ± 0.01 at an absorbance of 734 nm, and the ABTS working solution was obtained. An amount of 0.5 mL of BRE sample solutions with different concentrations (25, 50, 100, 200, 400, 600, and 800 mg/L) were accurately prepared and uniformly mixed with 3.0 mL of ABTS working solution. After 10 min of light avoidance reaction, the absorbance was measured at 734 nm and recorded as A_1_. According to the above method, 0.5 mL of BRE sample solutions with different concentrations (25, 50, 100, 200, 400, 600, and 800 mg/L) was uniformly mixed with 3.0 mL of anhydrous ethanol. The absorbance was measured at 734 nm and recorded as A_2_; 0.5 mL of 50% ethanol was uniformly mixed with 3.0 mL of ABTS working solution. The absorbance was measured at 734 nm and recorded as A_3_. Vitamin C (Vc) was used as the positive control [20]. The ABTS radical scavenging activity of the sample was calculated as follows:

(1) $$ABTS\;free\;radical\;scavenging\;rate\;(\%)=(1-\frac{A_{1}-A_{2}}{A_{3}})\times 100$$

#### 2.5.2. DPPH Radical Scavenging Test

An amount of 3.0 mL of BRE sample solutions with different concentrations (25, 50, 100, 200, 400, 600, and 800 mg/L) and 3.0 mL of DPPH (0.1 mmol/L) were accurately weighed and mixed. After the mixture was shaken, it was placed in the dark at 37 °C for 30 min. The absorbance was measured at 517 nm and recorded as A_1_. According to the above method, 3.0 mL of BRE sample solutions with different concentrations (25, 50, 100, 200, 400, 600, and 800 mg/L) and 3.0 mL of anhydrous ethanol were accurately weighed and mixed, and the absorbance was measured and recorded as A_2_; 3.0 mL of 50% ethanol was uniformly mixed with 3.0 mL of DPPH solution, and the absorbance was measured at 517 nm and recorded as A_3_. Vc was used as the positive control [21]. The DPPH radical scavenging activity of the sample was calculated as follows:

(2) $$DPPH\;radical\;scavenging\;rate\;\left(\%\right)\;(1-\frac{A_{1}-A_{2}}{A_{3}})\times 100$$

#### 2.5.3. Hydroxyl Radical Scavenging Test

An amount of 1.0 mL of BRE sample solution with different concentrations (25, 50, 100, 200, 400, 600, and 800 mg/L), 0.3 mL of 8.0 mmol/L FeSO_4_ solution, 0.25 mL of 20 mmol/L H_2_O_2_ solution, and 1.0 mL of 3.0 mmol/L salicylic acid solution were mixed and heated in a 37 °C water bath for 30 min and then left to cool to room temperature. The mixture was centrifuged at 3000× *g* for 10 min, and the supernatant was taken to measure the absorbance at 510 nm, which was recorded as A_1_. According to the above method, 1.0 mL of BRE sample solutions with different concentrations (25, 50, 100, 200, 400, 600, and 800 mg/L) and 0.3 mL of FeSO_4_, 0.25 mL of H_2_O, and 1.0 mL of salicylic acid were mixed and heated, and their absorbance was measured and recorded as A_2_. After 1.0 mL of 50% ethanol, 0.3 mL of FeSO_4_, 0.25 mL of H_2_O_2_, and 1.0 mL of salicylic acid were mixed and heated, their absorbance was measured and recorded as A_3_. Vc was used as the positive control [22]. The hydroxyl radical scavenging activity of the sample was calculated as follows:

(3) $$Hydroxyl\;radical\;scavenging\;rate\;\left(\%\right)=(1-\frac{A_{1}}{A_{2}\cdot A_{3}})\times 100$$

#### 2.5.4. Determination of β-Carotene Bleaching Inhibition

An amount of 2 mL β-carotene chloroform solution (2 mg/mL) was placed in a conical flask, 400 mg Tween 80 and 40 mg LA were added, and then chloroform was removed by rotary evaporation at 40 °C. An amount of 100 mL distilled water was added and shaken violently to form an emulsion. An amount of 0.2 mL of BRE sample solution with different concentrations (25, 50, 100, 200, 400, 600, and 800 mg/L) and 4.8 mL of emulsion were evenly mixed and added into the colorimetric tube, and the control tube was replaced by anhydrous ethanol. An amount of 200 μL of the above prepared mixture was added to the 96-well plate, and the absorbance value was measured at 470 nm, which was recorded as the absorbance value at “0 min”. The colorimetric tube was placed in a constant-temperature water bath at 50 °C for 2 h, and the measured absorbance value was recorded as the absorbance value at “2 h”. Each sample was determined three times. Trolox was selected as the positive control [23]. The β-carotene bleaching inhibition rate of the sample was calculated as follows:

(4) $$\beta -carotene\;bleaching\;inhibition\;rate\;\left(\%\right)=\left(\frac{A_{2h}-A_{k2h}}{A_{0}-A_{k}}\right)\times 100\%$$

A_2h_ and A_0_ are the absorbance values of the sample at 2 h and 0 min, A_k_ is the blank value, and A_k2h_ is the absorbance value of the control tube at 2 h.

### 2.6. Effect of BRE on Free Radical-Induced Protein Oxidative Damage

#### 2.6.1. Effect of BRE on CuSO_4_/H_2_O_2_-Induced Protein Oxidation and Carbonylation

An amount of 400 μL BSA solution with a concentration of 4 mg/mL was added with 100 μL sample solutions with different concentrations (0, 25, 50, 100, 200, and 500 mg/L) and evenly mixed. An amount of 250 μL CuSO_4_ solution and H_2_O_2_ solution with concentrations of 2 mmol/L and 50 mmol/L were successively added to the above mixed solutions, with a total reaction volume of 1 mL. After being sealed, they were placed in a 37 °C water bath for 90 min. In the control group, H_2_O_2_ and CuSO_4_ solutions were not added, and the same volume of pH 7.4 phosphate buffer was used instead. After the reaction, the carbonyl content of BSA was detected by DNPH colorimetry, SDS-PAGE separated the protein with 10% separating gel, and the Coomassie brilliant blue R-250 staining method was used to detect the oxidative damage of BSA [24].

#### 2.6.2. Effects of *Boschnikia rossica* Extract on Protein Oxidation and Carbonylation Induced by AAPH

An amount of 400 μL of BSA solution with a concentration of 4 mg/mL was added with 100 μL of sample solutions with different concentrations (0, 25, 50, 100, 200, and 500 mg/L) and evenly mixed. An amount of 500 μL of AAPH solution with a concentration of 250 mmol/L was added, with a total reaction volume of 1 mL. The seal was placed in a 37 °C water bath for reaction for 4 h. The control group did not add an AAPH solution and was replaced with the same volume of pH 7.4 phosphate buffer. After the reaction, the carbonyl content of BSA was detected by DNPH colorimetry, SDS-PAGE separated the protein with 10% separating gel, and the Coomassie brilliant blue R-250 staining method was used to detect the oxidative damage of BSA [24].

### 2.7. Effect of BRE on Free Radical-Induced Lipid Peroxidation

#### 2.7.1. Effect of BRE on FeSO_4_-Induced Oxidation of LA

An amount of 500 μL of LA with a concentration of 2 mmol/L was taken, and 100 μL of sample solutions with different concentrations (0, 25, 50, 100, 200, and 500 mg/L) was added. After mixing, 400 μL of FeSO_4_ solution with a concentration of 250 mmol/L was added, and the total reaction volume was 1 mL. After sealing, the reaction was conducted in a water bath at 37 °C for 24 h without light. After the reaction, the lipid oxidation level of LA was expressed by the content of MDA and conjugated dienes [25]. A UV–VIS spectrophotometer determined the absorbance of the sample at 233 nm, the conjugated diene content was calculated according to the molar absorptivity of conjugated dienes 2.8 × 10^4^ L/(mol·cm), and the results were expressed in μmol/L. The MDA content in the sample was determined using an MDA assay kit.

#### 2.7.2. Effect of BRE on Oxidation of LA Induced by AMVN

An amount of 500 μL of LA with a concentration of 2 mmol/L was taken, and 100 μL of sample solutions with different concentrations (0, 25, 50, 100, 200, and 500 mg/L) was added. After being mixed, 400 μL of AMVN solution with a concentration of 2.5 mmol/L (dissolved in methanol) was added, and the total reaction volume was 1 mL. After being sealed, the reaction was carried out in a water bath at 37 °C for 12 h without light. After the reaction, the lipid oxidation level of LA was evaluated by the content of MDA and conjugated dienes [25]. The determination method for conjugated dienes and MDA is the same as in Section 2.7.1.

### 2.8. The Effect of BRE on Free Radical-Induced DNA Oxidative Damage

#### 2.8.1. The Effect of BRE on AAPH-Induced DNA Oxidative Damage

An amount of 10 μL of BRE solutions with different concentrations (0, 25, 50, 100, 200, and 500 mg/L) was uniformly mixed with 5 μL of hsDNA (final mass concentration of 2 mg/mL), pBR322 DNA (final mass concentration of 80 ng/μL), and 5 μL of AAPH solution, with a final concentration of 25 mmol/L. The mixture was placed in a 37 °C constant-temperature water bath for 1 h to detect the degree of DNA oxidative damage. The blank control group was treated with the same volume of phosphate buffer solution instead of AAPH solution. In contrast, the oxidation model control group was treated with the same volume of phosphate buffer solution instead of BRE solution [2]. Then, 1% agarose gel was prepared, and electrophoresis was carried out for 30 min. After electrophoresis, a chemiluminescence imaging system was used for imaging. Quantity One ^®^ 1D-4.6.6 software was used to analyze the DNA band intensity of the pBR322 plasmid.

#### 2.8.2. Effect of BRE on CuSO_4_/H_2_O_2_-Induced DNA Oxidative Damage

The processing methods for hsDNA and pBR322 DNA were the same as in Section 2.8.1. CuSO_4_ and H_2_O_2_ solutions with final concentrations of 0.1 mmol/L and 1 mmol/L were added to a mixture of samples, hsDNA, and pBR322 DNA at different concentrations. The blank control group was treated with the same volume of phosphate buffer solution instead of CuSO_4_ and H_2_O solutions. In contrast, the oxidation model control group was treated with the same volume of phosphate buffer solution instead of the sample solution. After mixing, the mixture was placed in a constant-temperature water bath at 37 °C for 90 min to detect the degree of DNA oxidative damage [24]. The detection method for the degree of DNA oxidative damage was the same as that in Section 2.8.1.

#### 2.8.3. Determination of hsDNA Oxidative Damage

The level of hsDNA oxidative damage was measured using an MDA detection kit. The inhibition rate of BRE on AAPH- and CuSO_4_/H_2_O_2_-induced MDA production was calculated as follows:

(5) $$MDA\;generation\;inhibition\;rate\;\left(\%\right)=(1-\frac{A_{2}-A_{0}}{A_{1}-A_{0}})\times 100$$

A_0_ is the absorbance of the blank group (without inducer and sample solution); A_1_ is the absorbance of the induction group; and A_2_ is the absorbance after adding both the sample and the inducer simultaneously.

#### 2.8.4. DNA Agarose Gel Electrophoresis

The treated DNA reaction solution was mixed evenly with 2 μL sample loading buffer and then added to the agarose gel for the electrophoresis test. The electrophoresis voltage was 120 V, and the time was 30 min. After electrophoresis, the CHEMIDOC XRS gel imaging system was used to image the gel and Quantity One-4.6.6 software was used to conduct a semi-quantitative analysis of bands.

### 2.9. BRE Component Determination

LC-MS was used to determine the components of BRE. The specific method of LC-MS can be found in Supplementary Materials Document S1 [26,27,28,29,30].

### 2.10. Statistical Analysis

All experiments were repeated at least three times, and the results are presented as mean ± standard deviation. GraphPad Prism 10 was used for plotting and analysis. ****, ***, **, *, and ns, respectively, represent *p* < 0.0001, *p* < 0.001, *p* < 0.01, *p* < 0.05, and *p* > 0.05.

## 3. Results and Discussion

### 3.1. Optimization of Separation Conditions for HPLC

**Figure 1:**
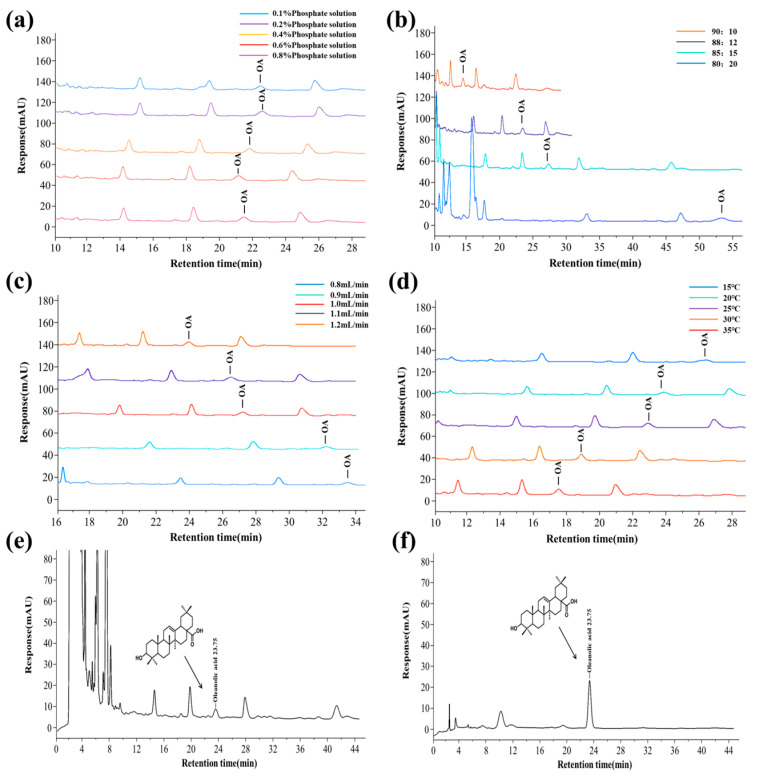
The effect of different chromatographic conditions on the peak time and separation of OA in BRE, as well as the optimized chromatograms of the sample and standard. (**a**) The influence of different phosphate buffer solutions; (**b**) the impact of different flow matching ratios; (**c**) the impact of different flow velocities; (**d**) the influence of different column temperatures; (**e**) chromatogram of *Boschnikia rossica* sample; and (**f**) chromatogram of oleanolic acid (OA) standard solution.

The chromatographic mobile phase was optimized to shorten the sample analysis time and reduce the interference of impurity components. Different concentrations of phosphate buffer solutions had no significant effect on OA’s peak time, peak area, or separation efficiency (Figure 1a). Compared with 0.1%, 0.2%, 0.6%, and 0.8% phosphoric acid solutions, 0.4% phosphoric acid solution as the mobile phase had a symmetrical and sharp peak shape, significantly improved sensitivity, and could effectively suppress impurity interference. Moreover, the pH value of 0.4% phosphoric acid solution matched the tolerance range of the chromatographic column (pH ≥ 2), which can effectively avoid column efficiency decline or column bed collapse caused by strong acidic conditions, significantly prolong the service life of the chromatographic column, and reduce maintenance costs. The chromatographic column’s peak shape, sensitivity, and service life were considered, and a 0.4% phosphoric acid aqueous solution was selected as the mobile phase.

The mobile phase ratio’s effect on OA retention time was significant (Figure 1b). When the mobile phase ratio increased from 80/20 to 90/10, OA’s retention time advanced. When the flow matching ratio was 85/15, the target peak had a reasonable peak time and a good separation effect from adjacent peaks. Therefore, 85/15 (*v*/*v*) was selected as the optimal flow matching ratio.

When the flow rate was low, the retention time of OA was prolonged, the analysis time was prolonged, and OA could not be well separated from adjacent peaks. When the flow rate was high, OA’s retention and analysis time were shortened, and OA could be well separated from adjacent peaks (Figure 1c). When the flow rate was 1.2 mL/min, the target peak could be well separated from adjacent peaks. When the flow rate exceeded 1.2 mL/min, the column pressure was too high, and the chromatographic column was severely damaged. Therefore, the optimal flow rate was chosen as 1.2 mL/min.

Raising the column temperature can reduce the mobile phase’s viscosity, improve mass transfer efficiency, and shorten analysis time. However, a too high column temperature was not conducive to separating OA and affected the service life of the chromatographic column. The column temperature of the chromatographic column was optimized, and the improvement in the separation degree of the target peak by column temperature at 15 °C, 20 °C, 25 °C, 30 °C, and 35 °C was investigated. As the column temperature increased, the retention time of OA shortened (Figure 1d). When the column temperature was 15 °C and 20 °C, the separation effect of OA from adjacent impurity peaks was better. Compared to a column temperature of 15 °C, the peak shape was better at a column temperature of 20 °C, and the detection efficiency was higher, meeting the analysis requirements. Therefore, considering all factors, a column temperature of 20 °C was selected.

### 3.2. Methodological Investigation and Sample Content Determination of RP-HPLC

Optimizing the chromatographic separation conditions obtained the optimal detection conditions for OA. The mobile phase was a methanol/ 0.4% phosphoric acid aqueous solution, with a mobile phase ratio of 85:15 (*v*/*v*), flow rate of 1.2 mL/min, column temperature of 20 °C, and detection wavelength of 220 nm. The chromatograms of the sample and standard product of OA from *Boschnikia rossica* are shown in Figure 1e,f. The content of OA in BRE was 0.358 mg/g.

#### 3.2.1. Linear Range, Correlation Coefficient, and Detection Limit

The mass concentration of OA was linearly related to its corresponding peak area within the range of 100–800 mg/L. The standard curve was Y (peak area) = 2.2405 X (equivalent concentration of OA) + 2.6461 (R^2^ = 0.9994). The method’s LOD was 1.92 mg/L, and the LOQ was 6.4 mg/L.

#### 3.2.2. Stability, Repeatability, Precision, and Spiked Recovery Experimental Results

The results of the repeatability experiment are shown in Table 1, with an RSD of 1.45%, indicating that this determination method had good repeatability and meets the analysis requirements. The stability results are shown in Table 2, and there was no significant change in the chromatographic peak area of OA, with an RSD of 1.21%, indicating that the test solution was stable within 24 h. The precision and spiked recovery experimental results are shown in Table 3. OA’s average spiked recovery rate was 98.88~101.46%, and the RSD was less than 5%, indicating that this method had high accuracy and met the analytical requirements.

### 3.3. BRE In Vitro Antioxidant Activity

#### 3.3.1. BRE’s Free Radical Scavenging Ability

**Figure 2:**
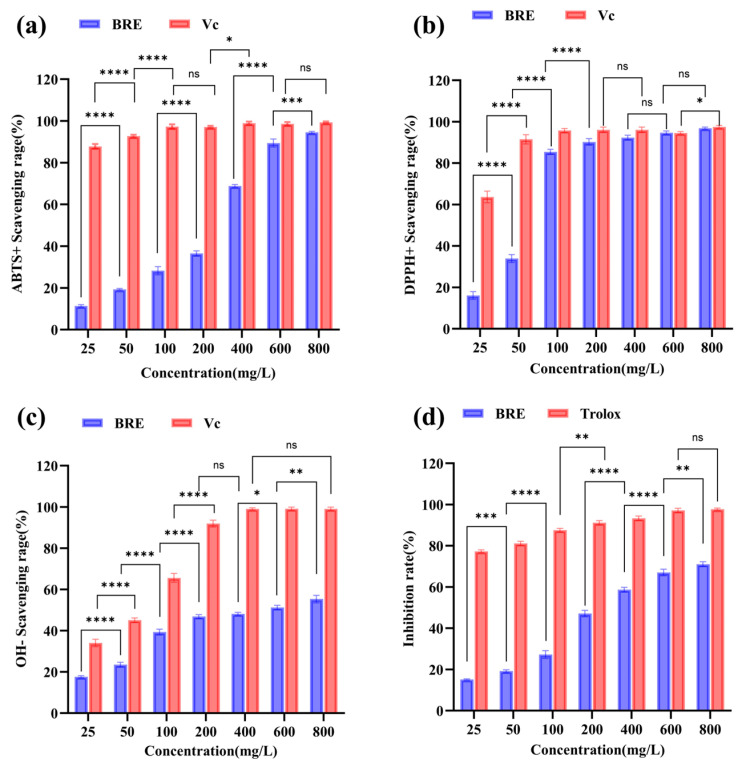
In vitro antioxidant activity of different concentrations of BRE. (**a**) ABTS radical scavenging ability; (**b**) DPPH radical scavenging ability; (**c**) •OH radical scavenging ability; and (**d**) β-carotene bleaching inhibition ability. ****, ***, **, *, and ns, respectively, represent *p* < 0.0001, *p* < 0.001, *p* < 0.01, *p* < 0.05, and *p* > 0.05.

Oxidative stress is considered an important factor leading to aging and disease [31]. In order to comprehensively evaluate the antioxidant capacity of BRE, the in vitro antioxidant activity of BRE was investigated through ABTS, DPPH, and hydroxyl radical scavenging assays. As shown in Figure 2a, the ABTS radical scavenging rate increased with the increase in BRE mass concentration, and when the BRE concentration was 800 mg/L, the ABTS radical scavenging rate was 95.12% (*p* < 0.05), with an IC_50_ of 224.32 mg/L. It was indicated that BRE had a strong ABTS radical scavenging ability. DPPH is a purple radical reduced to 2,2-diphenyl-1-picrylhydrazine when an antioxidant accepts an electron or hydrogen radical. The sensitivity of this radical was sufficient to detect the antioxidant activity of low-concentration extracts [32]. As shown in Figure 2b, the DPPH radical scavenging rate also increased with increased BRE mass concentration. The scavenging rate of 400 mg/L BRE could reach 93.06% (*p* < 0.05), with an IC_50_ of 58.43 mg/L. The above results indicated that BRE also had a strong scavenging ability against DPPH radicals.

The most active and common hydroxyl radicals produced endogenously during aerobic metabolism are considered highly destructive substances that can cause DNA damage and chain breakage, leading to cancer, mutations, and cell toxicity [33]. Finding natural molecules with high scavenging activity against these free radicals is significant. As shown in Figure 2c, the IC_50_ of BRE for scavenging hydroxyl radicals was 432.21 mg/L, and the hydroxyl radical scavenging rate of 800 mg/L BRE was 55.3% (*p* < 0.05). The scavenging ability of BRE for free radicals was significantly positively correlated, and the scavenging rate of free radicals increased significantly with the sample mass concentration. These results were consistent with previous research findings; the ability of plant extracts to scavenge free radicals increased with the extract concentration [34,35]. BRE’s significant free radical scavenging ability may be related to its active ingredients. Through LC-MS analysis of BRE, 17 main components were obtained and are presented in Table S1. It contained triterpenoids such as OA and ursolic acid, which have been shown to achieve antioxidant function by inhibiting ROS production and regulating superoxide dismutase (SOD) and glutathione (GSH) Px enzyme activity [13,36]. This was related to the unique molecular structure of triterpenoids. Firstly, multiple hydroxyl groups (especially the C3 hydroxyl group) in triterpenoid molecules could directly neutralize hydroxyl radicals (•OH) and peroxide radicals (ROO•) by providing hydrogen atoms (H^+^), blocking the chain reaction of free radicals. Secondly, the double bonds in the pentacyclic triterpenoid skeleton form a conjugated system, which stabilizes free radical intermediates through electron delocalization, prolongs the persistence of antioxidant activity, and disperses free radical energy to reduce attacks on biomolecules such as DNA and proteins. In addition, its hydrophobic structure endows it with lipophilic characteristics, making it easy to embed into the hydrophobic regions of cell membranes or lipoproteins. It protects the biofilm system from oxidative damage by inhibiting lipid peroxidation reactions (such as reducing the production of malondialdehyde (MDA)). Phenylethanoid glycosides, such as salidroside, often exert antioxidant effects through pathways such as scavenging free radicals and inhibiting lipid peroxidation due to their characteristic structure of benzyl and glycosidic bonds [37]. There were also flavonoids, phenols, and other substances in BRE, with total phenol, total flavonoid, and total triterpene contents of 46.83 mg/g, 6.94 mg/g, and 25.66 mg/g, respectively (Table S2). Together, they form the multi-target antioxidant network of BRE and synergistically exert antioxidant effects.

#### 3.3.2. Inhibition Ability of β-Carotene Bleaching

β-carotene is a polyene orange pigment that easily fades when oxidized. The LA in the emulsion will automatically oxidize, and the generated free radicals can react with β-carotene, causing the color of β-carotene to decay. Antioxidants can slow down the rate of color decay of β-carotene [38]. As shown in Figure 2d, BRE had a certain inhibitory effect on β-carotene bleaching within the concentration range of 100–800 mg/L, and the inhibition rate increased with the increase in concentration. When the sample concentration was 800 mg/L, the inhibition rate of BRE on β-carotene bleaching reached 71.11% (*p* < 0.05). BRE had a strong inhibitory effect on β-carotene bleaching, which may result from its active ingredients effectively maintaining the color stability of β-carotene by blocking the chain reaction of lipophilic free radicals. This has important application value in the antioxidant preservation of high-fat foods such as functional oils and dairy products.

### 3.4. The Effect of BRE on Free Radical-Induced Protein Oxidative Damage

#### 3.4.1. The Effect of BRE on AAPH-Induced BSA Oxidative Damage

**Figure 3:**
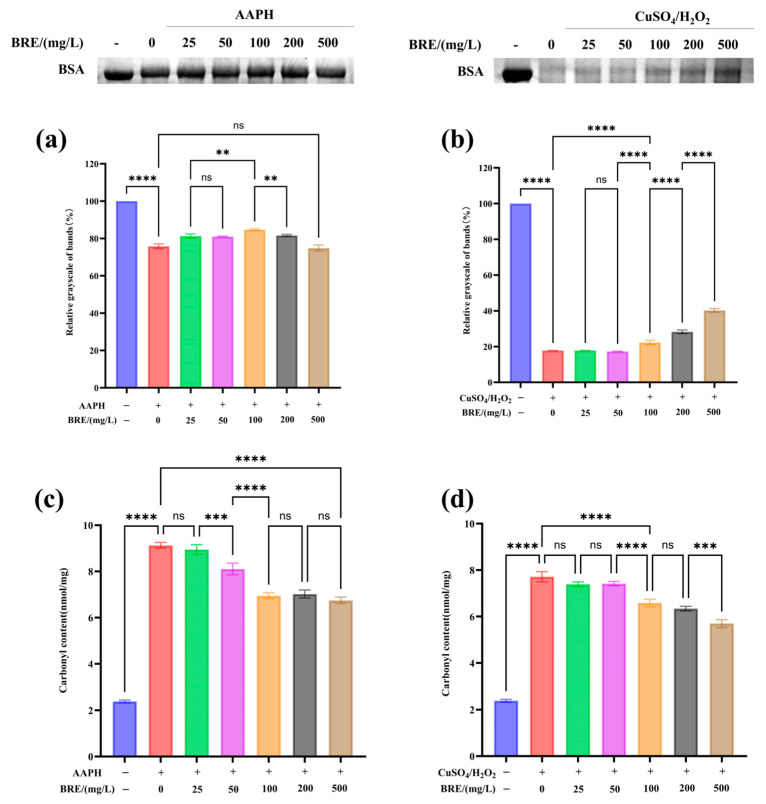
Protective effect of BRE on AAPH- and CuSO_4_/H_2_O_2_-induced BSA oxidative damage, (**a**) protective effect of BRE on AAPH-induced BSA oxidative damage; (**b**) BRE has a protective effect against CuSO_4_/H_2_O_2_-induced BSA oxidative damage; (**c**) BRE induces changes in BSA carbonyl content in AAPH; and (**d**) BRE induces changes in BSA carbonyl content with CuSO_4_/H_2_O_2_. ****, ***, **, and ns, respectively, represent *p* < 0.0001, *p* < 0.001, *p* < 0.01, and *p* > 0.05.

Free radicals can cause oxidative damage to proteins, lipids, and DNA. The presence of antioxidants can block the oxidation chain of free radicals, thereby inhibiting protein, DNA, and lipid oxidation [39]. 2,2-Azobis (2-methylpropylimidazole) dihydrochloride (AAPH) is an azide compound and a recognized free radical inducer. At 37 °C and pH 7.0, AAPH decomposes to generate nitrogen and carbon free radicals, which can further react with oxygen to generate reactive oxygen species. When reactive oxygen species attack proteins, it can lead to peptide bond cleavage [40]. The generation of peroxide free radicals through the AAPH system induces oxidative damage to proteins, and the protective effect of BRE on protein oxidative damage was investigated. As shown in Figure 3a, SDS-PAGE results indicated that BRE could significantly inhibit AAPH-induced BSA oxidative degradation, showing concentration dependence in the 25–100 mg/L range, with the strongest effect at 100 mg/L. As the sample mass concentration increased (200–500 mg/L), the inhibitory effect on AAPH-induced BSA oxidative damage weakened. This result might be because low concentrations of BRE could effectively eliminate nitrogen and carbon free radicals generated by AAPH decomposition, reduce the production of reactive oxygen species, and thus reduce protein oxidative damage. However, at high concentrations, active ingredients in the extract (such as flavonoids, phenols, and terpenes) may undergo self-oxidation due to excessive exposure to oxidative environments, generating intermediate products with oxidative activity, such as semiquinone free radicals and peroxides, which further trigger chain reactions and exacerbate protein oxidative damage [41].

#### 3.4.2. The Effect of BRE on CuSO_4_/H_2_O_2_-Induced BSA Oxidative Damage

Metal ions such as Fe^2+^, Fe^3+^, Cu^2+^, Mn^2+^, and Ni^2+^ can catalyze the decomposition of H_2_O_2_, producing hydroxyl radicals with higher oxidation activity, which can react with all amino acids [42]. As shown in Figure 3b, the degree of BSA oxidative damage induced by CuSO_4_/H_2_O_2_ significantly increased. CuSO_4_/H_2_O_2_ generated hydroxyl radicals through the Fenton reaction, which induced BSA to undergo oxidative degradation, decreasing protein band grayscale. After treatment with 500 mg/L BRE, the degree of BSA oxidative degradation was significantly reduced (*p* < 0.05). This result was consistent with the hydroxyl radical scavenging test results of BRE mentioned above. Cu^2+^ can catalyze the decomposition of H_2_O_2_ to produce many hydroxyl radicals. At the same time, BRE can slow down the oxidative damage caused by free radicals to BSA by scavenging the hydroxyl radicals generated by the reaction, which is consistent with the research results of Zhike et al. [43]. BRE exhibits different effects in the AAPH and CuSO_4_/H_2_O_2_ reaction systems, possibly due to the different properties of free radicals and their binding sites on biomolecules in the two systems.

#### 3.4.3. Effect of BRE on AAPH- and CuSO_4_/H_2_O_2_-Induced Carbonylation of BSA

Protein carbonylation is an important indicator for measuring protein oxidative damage [44]. The degree of carbonylation of BSA itself was very low, and significant carbonylation modification occurs under the action of CuSO_4_/H_2_O_2_ and AAPH. The structural carbonylation change will further cause changes in protein function [45]. As shown in Figure 3c,d, under the action of CuSO_4_/H_2_O_2_ and AAPH, the carbonyl content of BSA significantly increased. The carbonyl content in the CuSO_4_/H_2_O_2_ induction system increased from 2.39 nmol/mg to 7.71 nmol/mg (*p* < 0.05), and the carbonyl content in the AAPH induction system increased from 2.39 nmol/mg to 9.13 nmol/mg (*p* < 0.05). As the BRE concentration increased, the BSA’s carbonyl content induced by AAPH and CuSO_4_/H_2_O_2_ significantly decreased. BRE at 500 mg/L reduced the carbonyl content of BSA induced by AAPH and CuSO_4_/H_2_O_2_ to 6.73 nmol/mg and 5.7 nmol/mg, respectively (*p* < 0.05). Zhike et al. found that BPCC can significantly inhibit AAPH- and CuSO_4_/H_2_O_2_-induced BSA carbonylation modification due to its ability to scavenge free radicals and chelate metal ions [43]. In this study, BRE also significantly inhibited free radical-induced BSA carbonylation modification, which may be due to its ability to inhibit BSA carbonylation modification by clearing hydroxyl and alkoxy radicals generated by CuSO_4_/H_2_O_2_ and AAPH reaction systems, thereby suppressing protein oxidative damage.

### 3.5. The Effect of BRE on Free Radical-Induced Lipid Peroxidation

#### 3.5.1. The Effect of BRE on AMVN-Induced Lipid Peroxidation

Linoleic acid, a typical polyunsaturated fatty acid, has a diallyl structure, making it highly susceptible to lipid peroxidation through free radical chain reactions. During lipid peroxidation, oxidation products such as 4-hydroxynonenal (4-HNE), linoleic acid hydroperoxide (LOOH), and malondialdehyde (MDA) are formed, which can react with TBAR to generate TBARS. Its content is one of the important indicators to measure the degree of lipid peroxidation [46].

**Figure 4:**
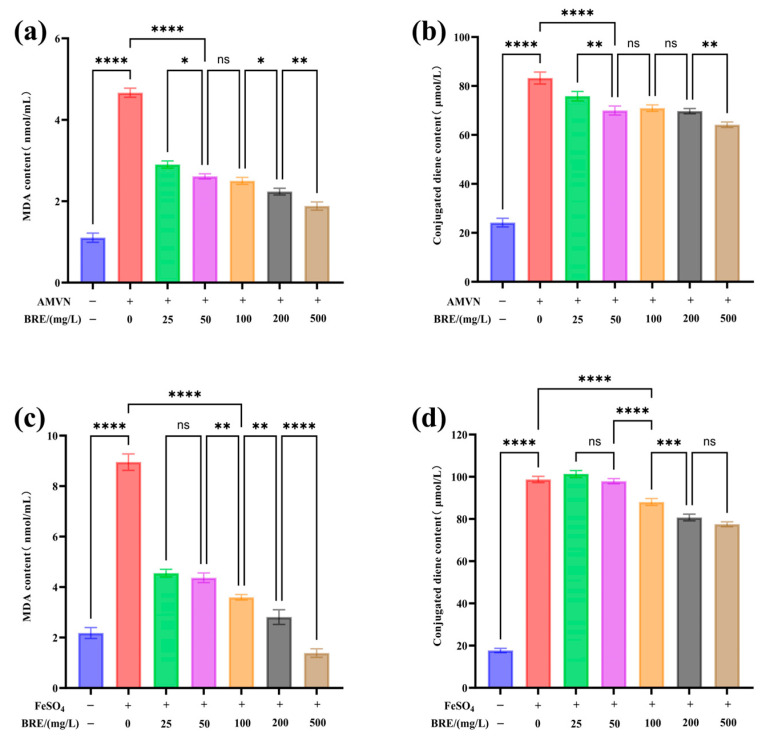
The effect of BRE on FeSO_4_- and AMVN-induced LA peroxidation, (**a**) BRE-induced changes in MDA content by AMVN; (**b**) BRE induces changes in conjugated diene content in AMVN; (**c**) BRE induces changes in MDA content in FeSO_4_; and (**d**) BRE induces changes in conjugated diene content induced by FeSO_4_. ****, ***, **, *, and ns, respectively, represent *p* < 0.0001, *p* < 0.001, *p* < 0.01, *p* < 0.05, and *p* > 0.05.

Figure 4a,b show that the control group had a lower degree of LA oxidation, with MDA and conjugated diene contents of 1.1 nmol/mL and 25.36 μmol/L, respectively. Under the induction of AMVN, the model group LA underwent lipid oxidation, and the MDA and conjugated diene contents produced by LA oxidation significantly increased to 4.66 nmol/mL and 82.36 μmol/L (*p* < 0.05). The significant difference confirms that AMVN effectively initiates a free radical chain reaction by generating alkoxyl radicals by pyrolysis, leading to a typical lipid peroxidation process in LA. The MDA and conjugated diene content produced by LA oxidation significantly decreased with increased BRE concentration. The MDA and conjugated diene content in the 500 mg/L BRE group decreased to 1.85 nmol/mL and 64.54 μmol/L, respectively (*p* < 0.05). The MDA level was close to the basal oxidation level, indicating that BRE can significantly inhibit the generation of MDA and conjugated dienes during AMVN-induced lipid oxidation.

#### 3.5.2. The Effect of BRE on FeSO_4_-Induced Lipid Peroxidation

Figure 4c,d show that in the control group, LA produced MDA and conjugated diene contents of 2.18 nmol/mL and 18.67 μmol/L, respectively. However, after induction with FeSO_4_, LA underwent lipid oxidation, resulting in a significant increase in MDA production (8.98 nmol/mL) and conjugated diene content (96.45 μmol/L) (*p* < 0.05), indicating the successful establishment of the model. In the FeSO_4_-induced oxidative damage system, different concentrations of BRE affect lipid peroxidation levels to varying degrees. When the concentration of BRE was 500 mg/L, the MDA and conjugated diene content decreased to 1.38 nmol/mL and 77.52 μmol/L, respectively (*p* < 0.05), indicating that BRE can also effectively inhibit FeSO_4_-induced LA oxidation.

FeSO_4_- and AMVN-induced lipid peroxidation results showed that BRE exhibited significant antioxidant activity against two different mechanisms of oxidative damage. In the AMVN model, intervention with 500 mg/L extract reduced the production of MDA and conjugated dienes by 60.3% and 21.64%, respectively, compared to the model group, with MDA levels restored to a near-basal oxidative state. In the FeSO_4_ model, the inhibition rates of 500 mg/L extract on MDA and conjugated dienes reached 84.63% and 19.6%, respectively. It was worth noting that the inhibitory efficiency of BRE on MDA in both models was significantly higher than that on conjugated dienes, which might be related to its multi-target antioxidant mechanism. On the one hand, the phenolic or flavonoid components in BRE can inhibit the initiation of the oxidative chain reaction by directly clearing alkoxyl radicals or chelating Fe^2+^ to block the Fenton reaction [47]. On the other hand, it might terminate the chain propagation of lipid free radicals (L•/LOO•) through hydrogen supply, thereby reducing the accumulation of intermediate products such as LOOH and the subsequent generation of MDA [48].

### 3.6. Effect of BRE on Free Radical-Induced DNA Oxidative Damage

#### 3.6.1. The Effect of BRE on AAPH- and CuSO_4_/H_2_O_2_-Induced Oxidative Damage of pBR322 DNA

**Figure 5:**
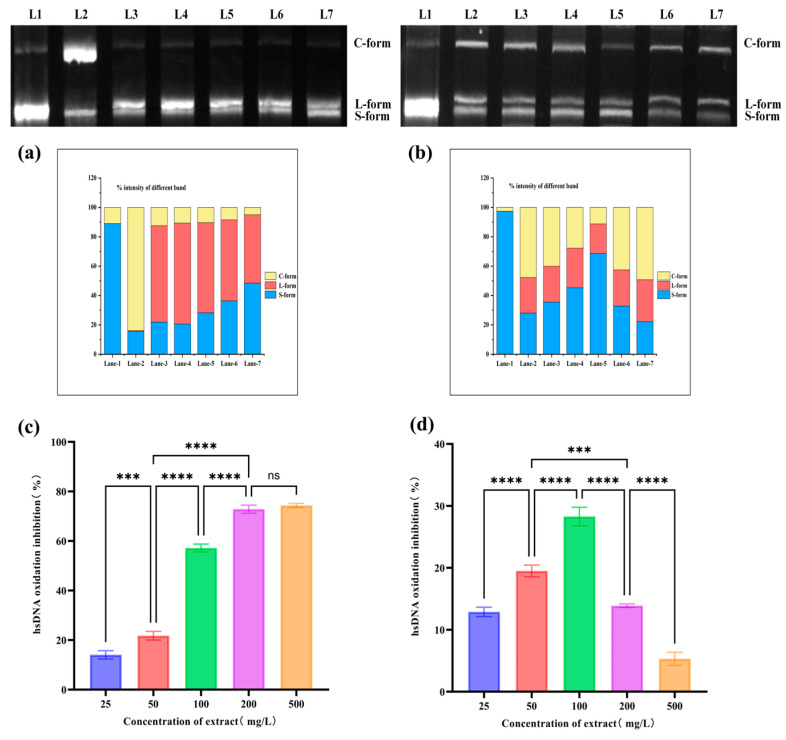
Effects of BRE on AAPH- and CuSO_4_/H_2_O_2_-induced oxidative damage to pBR322 DNA and hsDNA. (**a**) The inhibitory effect of BRE on AAPH-induced oxidation of pBR322 DNA; (**b**) the inhibitory effect of BRE on CuSO_4_/H_2_O_2_-induced oxidation of pBR322 DNA. Lane-1: pBR322 DNA; Lane-2: pBR322 DNA + AAPH/CuSO_4_/H_2_O_2_; Lane-3: pBR322 DNA + AAPH/CuSO_4_/H_2_O_2_ + 25 mg/L BRE; Lane-4: pBR322 DNA + AAPH/CuSO_4_/H_2_O_2_ + 50 mg/L BRE; Lane-5: pBR322 DNA + AAPH/CuSO_4_/H_2_O_2_ + 100 mg/L BRE; Lane-6: pBR322 DNA + AAPH/CuSO_4_/H_2_O_2_ + 200 mg/L BRE; and Lane-7: pBR322 DNA + AAPH/CuSO_4_/H_2_O_2_ + 500 mg/L BRE. (**c**) The inhibitory effect of BRE on AAPH-induced hsDNA oxidation; (**d**) the inhibitory effect of BRE on hsDNA oxidation induced by CuSO_4_/H_2_O_2_. ****, ***, and ns, respectively, represent *p* < 0.0001, *p* < 0.001 and *p* > 0.05.

DNA is one of the most important target molecules for reactive oxygen species attacks. After being attacked by free radicals, DNA undergoes base modifications and strand breaks, and DNA oxidative damage is closely related to the occurrence and development of various diseases. Figure 5a,b show that almost all the blank group pBR322 DNA exists in a supercoiled structure. However, after induction with AAPH and CuSO_4_/H_2_O_2_, the amount of open-loop and linear pBR322 DNA significantly increased, indicating that the free radicals generated by the AAPH and CuSO_4_/H_2_O_2_ induction system caused oxidative damage to pBR322 DNA and disrupted its supercoiled structure.

With the increase in BRE concentration, the amount of open-loop DNA and linear DNA significantly decreased (*p* < 0.05) in the AAPH induction system, and the amount of supercoiled DNA increased accordingly. BRE at 500 mg/L could protect plasmid DNA from AAPH-induced oxidative damage. This indicated that BRE had a protective and reparative effect on AAPH-induced oxidative damage to DNA, consistent with the findings of Yates et al. [49]. In the AAPH-induced oxidation system, BRE exhibited a significant protective effect, possibly related to its active ingredients. These active ingredients synergistically form a multi-level defense mechanism. On the one hand, phenolic hydroxyl groups directly neutralize the RO• and ROO• radicals produced by AAPH through hydrogen atom transfer, blocking their oxidative attacks on the DNA phosphate backbone and deoxyribose. On the other hand, these components could inhibit metal-catalyzed secondary radical chain reactions by chelating trace metal ions (such as Fe^2^⁺/Cu^2^⁺) in the system. At the same time, they could embed into the lipid microenvironment around DNA through hydrophobic interactions, inhibit the generation of lipid peroxidation products, and indirectly reduce the additional damage of active aldehydes to DNA.

In the CuSO_4_/H_2_O_2_ induction system, the protective effect of BRE exhibits a biphasic effect. After incubation with 25–100 mg/L BRE, the linear and open-loop pBR322 DNA significantly decreased, while the amount of DNA with a supercoiled structure increased significantly in a concentration-dependent manner. The sample with a mass concentration of 100 mg/L showed the best protective effect against DNA oxidative damage, but as the concentration continued to increase, the amount of pBR322 DNA with an open-loop structure increased, and the amount of pBR322 DNA with a supercoiled structure decreased significantly. The weakened inhibitory effect of high-concentration BRE on DNA oxidation may be related to the reaction system between Cu^2+^ and H_2_O_2_. The principle of Cu^2+^ reacting with H_2_O_2_ to generate hydroxyl radicals is as follows: Cu^2+^ + H_2_O_2_ → Cu^+^ + HO_2_• + H^+^, H_2_O_2_ + Cu^+^ → OH• + OH^−^ + Cu^2+^. The reaction equation shows that the transition from Cu^2+^ to Cu^+^ is the rate-limiting step for generating hydroxyl radicals. Adding high concentrations of BRE might promote the transition from Cu^2+^ to Cu^+^, accelerate the production of hydroxyl radicals, weaken their protective effect on pBR322 DNA oxidation, and even promote DNA oxidative damage.

#### 3.6.2. The Effect of BRE on Oxidative Damage of hsDNA Induced by AAPH and CuSO_4_/H_2_O_2_

Free radicals attack DNA, producing MDA and many oxidative products. The degree of oxidative damage can be determined by measuring the absorbance of DNA oxidative products at 532 nm. Figure 5c,d show that hsDNA oxidative damage significantly increased after CuSO_4_/H_2_O_2_ and AAPH induction. As the concentration of BRE increased, its inhibitory effect on AAPH-induced DNA oxidative damage was enhanced, indicating that BRE could effectively inhibit AAPH-induced hsDNA oxidation and was concentration-dependent. BRE at 25–100 mg/L had a good protective effect on CuSO_4_/H_2_O_2_-induced hsDNA oxidation, with the best inhibitory effect observed at 100 mg/L. As the concentration continues to increase (200–500 mg/L), the protective effect of BRE on hsDNA oxidation weakens, consistent with the results of AAPH and CuSO_4_/H_2_O_2_-induced pBR322 DNA damage. Two models comprehensively evaluate DNA damage from two dimensions: structural damage and generation of oxidative products. The results of both dimensions jointly validate the protective effect of BRE on free radical-induced DNA oxidative damage.

## 4. Conclusions

This study established a method for analyzing the content of OA in BRE by HPLC and investigated the effect of different chromatographic conditions on eliminating matrix interference in BRE. Under optimal chromatographic conditions, the target peak had advantages such as a symmetrical peak shape, short separation time, and good separation effect. Standard addition experiments were performed to evaluate the matrix effect, yielding recoveries of 98.88–101.46% with RSD values below 5%, demonstrating minimal interference from the BRE matrix. The methodological investigation results indicate that all experiments meet the analytical requirements, and the method is simple, accurate, and has good reproducibility, which could provide a basis for effective quality control of OA in BRE. The results of in vitro antioxidant tests showed that BRE affects ABTS, DPPH, and hydroxyl radicals, has good scavenging ability, and has a strong inhibitory effect on β-carotene bleaching. In addition, BRE had significant inhibitory effects on protein oxidative degradation, carbonylation modification, lipid oxidation, and DNA oxidative damage induced by different free radicals. This study systematically evaluated the multi-target antioxidant activity of BRE, providing qualitative and quantitative evidence for the quality control and antioxidant activity of *Boschnikia rossica* and providing a scientific basis for further development of functional food from it.

## Figures and Tables

**Table 1:** Repeatability test results.

Serial Number	Peak Area (mAU·s)	Quantity Contained (mg/L)	Average Content (mg/L)	RSD/%
1	402.16	178.31	179.13	1.45
2	408.37	181.08		
3	397.65	176.31		
4	402.12	178.29		
5	400.17	177.43		
6	413.52	183.38		

**Table 2:** Stability test results.

Times/h	0	2	4	8	12	24	RSD/%
peak area (mAU·s)	412.73	405.85	410.21	399.75	402.16	403.78	1.21

**Table 3:** Precision and spiked recovery test results.

Serial Number	Quantity Contained (mg/L)	Marked Quantity(mg/L)	Measured Content (mg/L)	Recovery Rate/%	Average Recovery Rate/%	RSD/%
1	178.31	50	226.46	96.30%	98.88%	0.95
2	178.31	50	229.18	101.74%		
3	181.47	50	230.77	98.60%		
4	177.29	100	278.33	101.04%	101.46%	0.69
5	177.87	100	276.86	98.99%		
6	176.31	100	280.66	104.35%		
7	178.31	200	376.66	99.18%	100.95%	1.25
8	181.47	200	386.13	102.33%		
9	177.87	200	380.55	101.34%		

## Data Availability

The original contributions presented in the study are included in the article/Supplementary Materials. Further inquiries can be directed to the corresponding author.

## Supplementary Materials

The following supporting information can be downloaded at https://www.mdpi.com/article/10.3390/foods14101658/s1: Supplementary Document S1: LC-MS and determination of active ingredient content, Specific methods are detailed in references. Table S1. Identification of BRE components, Table S2. Total flavonoid, polyphenol, and triterpenoid content in the extract of BRE, Figure S1. Rutin standard curve, Figure S2. Gallic acid standard curve, Figure S3. Standard Curve of Oleanolic Acid; Supplementary Document S2: Gel raw image; Supplementary Document S3: Pictures of papers.
