# Supplementary material for: The Protective Effect of Boschnikia rossica Extract on Free Radical-Induced Oxidative Damage of Biomolecules and Establishment of a Method for Determining the Content of Oleanolic Acid
Source: Foods. 2025 May 8;14(10):1658. doi: 10.3390/foods14101658 (PMC12110839; doi:10.3390/foods14101658)
Supplement: Supplementary file 1 [file foods-14-01658-s001.zip › Supplementary Document S1-LC-MS and determination of active ingredient content.pdf]

LC-MS and determination of active ingredient content

1.1. Preparation of test solution

An appropriate amount of sample was accurately weighed in a 2 mL centrifuge tube, and 600 μL of methanol containing 2-chloro-L-phenylalanine (4 ppm) was added and vortexed for 30 s; The sample was placed in a tissue grinder and ground at 55 Hz for 60 s; Ultrasonic treatment at room temperature for 15 min; The supernatant was centrifuged at 12000 rpm and 4 ℃ for 10 min, filtered through a 0.22 μm membrane, and added to a detection bottle for LC-MS detection.

1.2. Liquid chromatography conditions

The LC analysis was performed on a Vanquish UHPLC System (Thermo Fisher Scientific, USA). Chromatography was carried out with an ACQUITY UPLC ® HSS T3 (2.1×100 mm, 1.8 μm) (Waters, Milford, MA, USA). The column maintained at 40 ℃. The flow rate and injection volume were set at 0.3 mL/min and 2 μL, respectively. For LC-ESI (+)-MS analysis, the mobile phases consisted of (B2) 0.1% formic acid in acetonitrile (v/v) and (A2) 0.1% formic acid in water (v/v). Separation was conducted under the following gradient: 0~1 min, 10% B2; 1~5 min, 10%~98% B2; 5~6.5 min, 98% B2; 6.5~6.6 min, 98%~10% B2; 6.6~8 min, 10% B2. For LC-ESI (-)-MS analysis, the analytes was carried out with (B3) acetonitrile and (A3) ammonium formate (5 mM). Separation was conducted under the following gradient: 0~1 min, 10% B3; 1~5 min, 10%~98% B3; 5~6.5 min, 98% B3; 6.5~6.6 min, 98%~10% B3; 6.6~8 min, 10% B3 [47]。

1.3. Mass spectrum conditions

Mass spectrometric detection of metabolites was performed on Q Exactive(Thermo Fisher Scientific, USA) with ESI ion source. Simultaneous MS1 and MS/MS (Full MS-ddMS2 mode, data-dependent MS/MS) acquisition was used. The parameters were as follows: sheath gas pressure, 40 arb; aux gas flow, 10 arb; spray voltage, 3.50 kV and -2.50 kV for ESI(+) and ESI(-), respectively; capillary temperature, 325 ℃; MS1 range, m/z 100-1000; MS1 resolving power, 70000 FWHM; number of data dependant scans per cycle, 10; MS/MS resolving power, 17500 3 FWHM; normalized collision energy, 30 eV; dynamic exclusion time, automatic [48].

Table S1. Identification of BRE components

serial number	major compound	m/z	RT/s	relative molecular mass	ppm	molecular formula
1	Salidroside	318.1541	120.8	300.1209	1.956	$C_{14}H_{20}O_7$
2	Syringin	390.1778	181.1	372.14203	4.996	$C_{17}H_{24}O_9$
3	Loganin	389.1375	205	390.15259	4.335	$C_{17}H_{26}O_{10}$
4	secologanin	345.1543	233.4	388.1369404	0.25154	$C_{17}H_{24}O_{10}$
5	Oleanolic acid	458.3951	399	456.7	9.023264	$C_{30}H_{48}O_3$
6	Ursolic acid	455.3491	448.1	456.3603	8.673	$C_{30}H_{48}O_3$
7	Corosolic acid	471.351	371.9	472.35524	6.652	$C_{30}H_{48}O_4$

8	Pinoresinol	357.1314	251.7	358.14163	8.274	$C_{20}H_{22}O_6$
9	Caffeic acid	163.0385	57.2	180.04226	2.872	$C_9H_8O_4$
10	Catechol	155.0351	44.9	110.0367776	0.766408	$C_6H_6O_2$
11	Luteolin	287.2005	447.6	286.04774	0.16	$C_{15}H_{10}O_6$
12	Naringenin	311.1645	144.2	272.0684702	5.532829	$C_{15}H_{12}O_5$
13	Kaempferide	299.0549	300.4	300.06339	4.042	$C_{16}H_{12}O_6$
14	Aucubin	327.1087	52.9	346.12638	0.505	$C_{15}H_{22}O_9$
15	(-)-lariciresinol	341.142	258.5	360.1572804	7.507197	$C_{20}H_{24}O_6$
16	Salicylic acid	137.0233	167.3	138.03169	8.146	$C_7H_6O_3$
17	Protocatechuic acid	153.0194	50.7	154.02661	0.45	$C_7H_6O_4$

1.4. Determination of total flavonoid content

3 mL of sample solution and 2.4 mL of 60% ethanol solution were sequentially taken into a 10 mL test tube. 0.3 mL of 5% $NaNO_2$ solution was added, shaken thoroughly, and allowed to stand for 5 min. 0.3 mL of 10% Al $(NO_3)_3$ solution was added, shaken well, and allowed to react for 6 min. 4 mL of 1 mol/L NaOH solution was added, shaken well, and left for 20 min. The absorbance of the sample was measured at 510 nm [49]. As shown in Figure S1, the standard curve obtained by plotting different concentrations of rutin standard solution is y=0.00589x+0.018544 (R^2=0.9996), and the result is expressed as the equivalent milligrams of rutin contained in the sample.

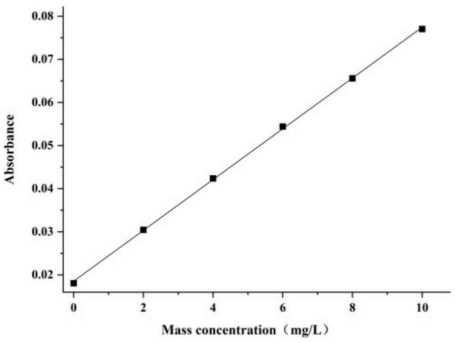

Figure S1. Rutin standard curve

1.5. Determination of total phenolic content

1 mL of moderately diluted sample solution was taken into 10 mL test tubes, and 1 mL of 1 mol/L phenol reagent, 3 mL of 7.5% Na_2CO_3 solution, and 5 mL of distilled water were added in sequence. After thorough mixing, they were placed in a 75 °C water bath for 10 min, cooled to room temperature with running water, and the absorbance was measured at 765 nm wavelength [50]. As shown in Figure S2, the standard curves obtained by plotting different concentrations of gallic acid standard solutions are as follows: y=0.00463x+0.00163（R^2＝0.9994）， The results are expressed in milligrams of gallic acid equivalent in the sample.

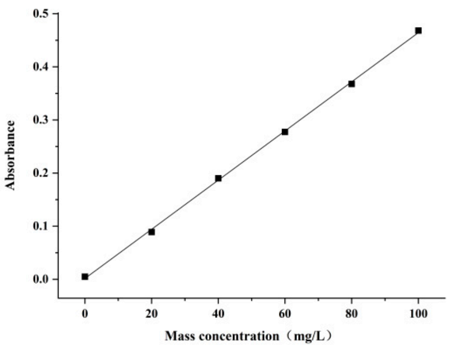

Figure S2. Gallic acid standard curve

1.6. Determination of total triterpenoid content

0.4 mL of sample solution was taken from a test tube, evaporated in a hot water bath, cooled to room temperature, and 0.4 mL of 5% vanillin glacial acetic acid and 1.6 mL of perchloric acid solution were added. They were then immersed in a 60 ℃ water bath for 15 min, cooled, and 5.0 mL of ethyl acetate was added. After mixing, they were allowed to stand for 15 min and the absorbance value was measured at 560 nm [51]. As shown in Figure S3, the standard curve y=0.00767x+0.06515 (R^2=0.9994) is used to calculate the total triterpenoid content, which is expressed in milligrams of equivalent oleanolic acid.

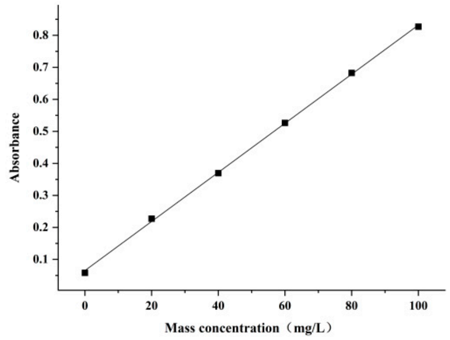

Figure S3. Standard Curve of Oleanolic Acid

1.7. Determination of Total Polyphenols, Flavonoids, and Triterpenes in BRE

The results of total flavonoids, total phenols, and total triterpenoids in the BRE were shown in Table S2.

Table S2. Total flavonoid, polyphenol, and triterpenoid content in the extract of BRE.

ingredient	linear regression equation	regression coefficient（R^2）	quantity contained（mg/g）
total flavonoids	y = 0.00589x+0.018544	0.9996	6.94
total phenol	y = 0.00463x+0.00163	0.9994	46.83
total triterpenes	y＝0.00767x+0.06515	0.9994	25.66
