# Supplementary material for: The Protective Effect of Boschnikia rossica Extract on Free Radical-Induced Oxidative Damage of Biomolecules and Establishment of a Method for Determining the Content of Oleanolic Acid
Source: Foods. 2025 May 8;14(10):1658. doi: 10.3390/foods14101658 (PMC12110839; doi:10.3390/foods14101658)
Supplement: Supplementary file 1 [file foods-14-01658-s001.zip › Supplementary Document S2-Gel Raw Image.pdf]

Supplementary Material S2

Supplementary Material S2 was the original images of Figures 3 and 5 in the manuscript.

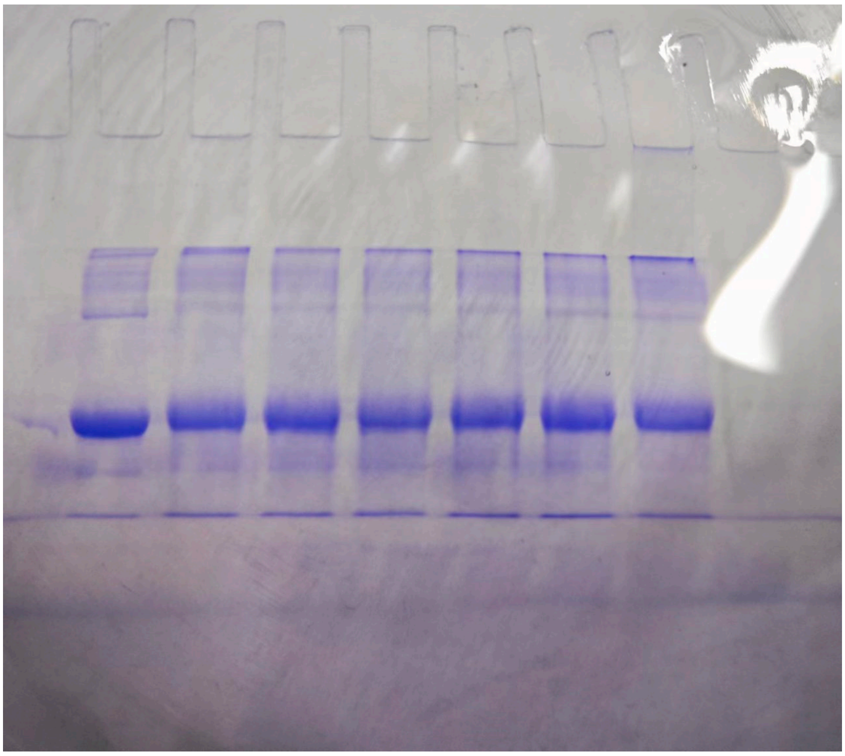

Figure. 3a

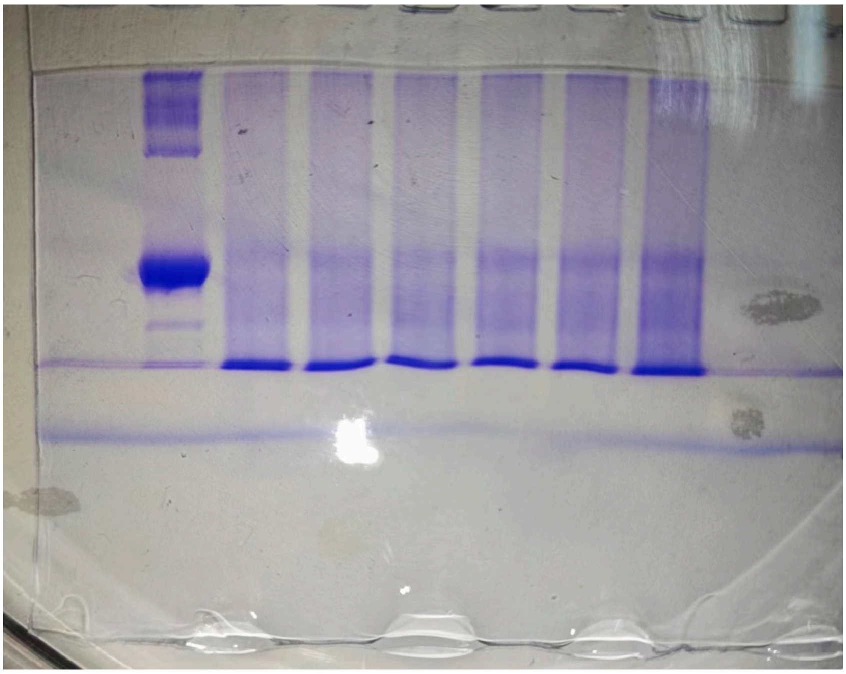

Figure. 3b

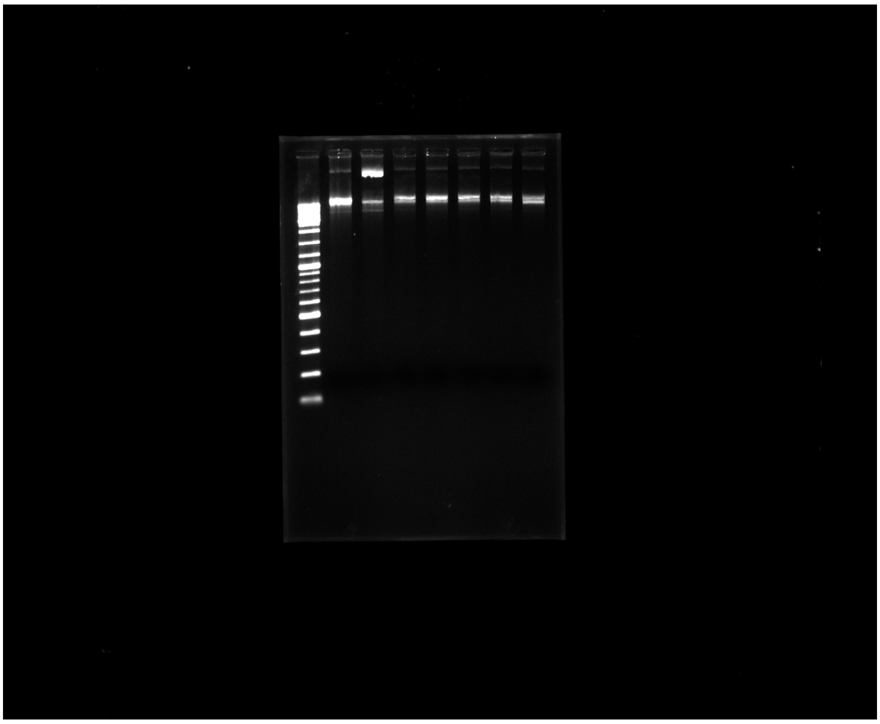

Figure. 5a

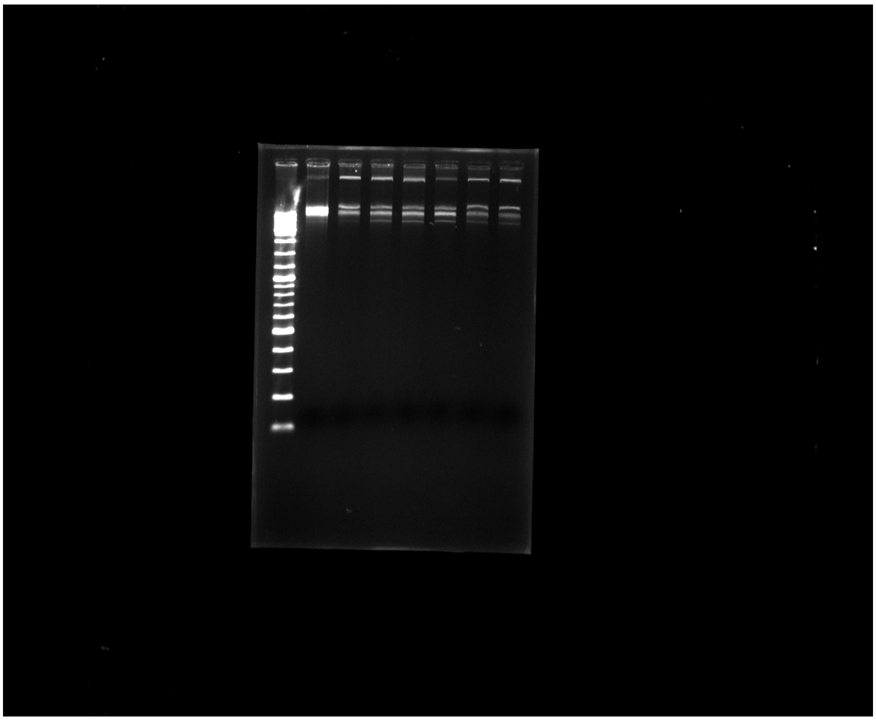

Figure. 5b
