# Supplementary material for: The Protective Effect of Boschnikia rossica Extract on Free Radical-Induced Oxidative Damage of Biomolecules and Establishment of a Method for Determining the Content of Oleanolic Acid
Source: Foods. 2025 May 8;14(10):1658. doi: 10.3390/foods14101658 (PMC12110839; doi:10.3390/foods14101658)
Supplement: Supplementary file 1 [file foods-14-01658-s001.zip › Supplementary Document S3-Pictures of papers.pdf]

Supplementary Material S3

1. This image is Figure 1 from the manuscript.

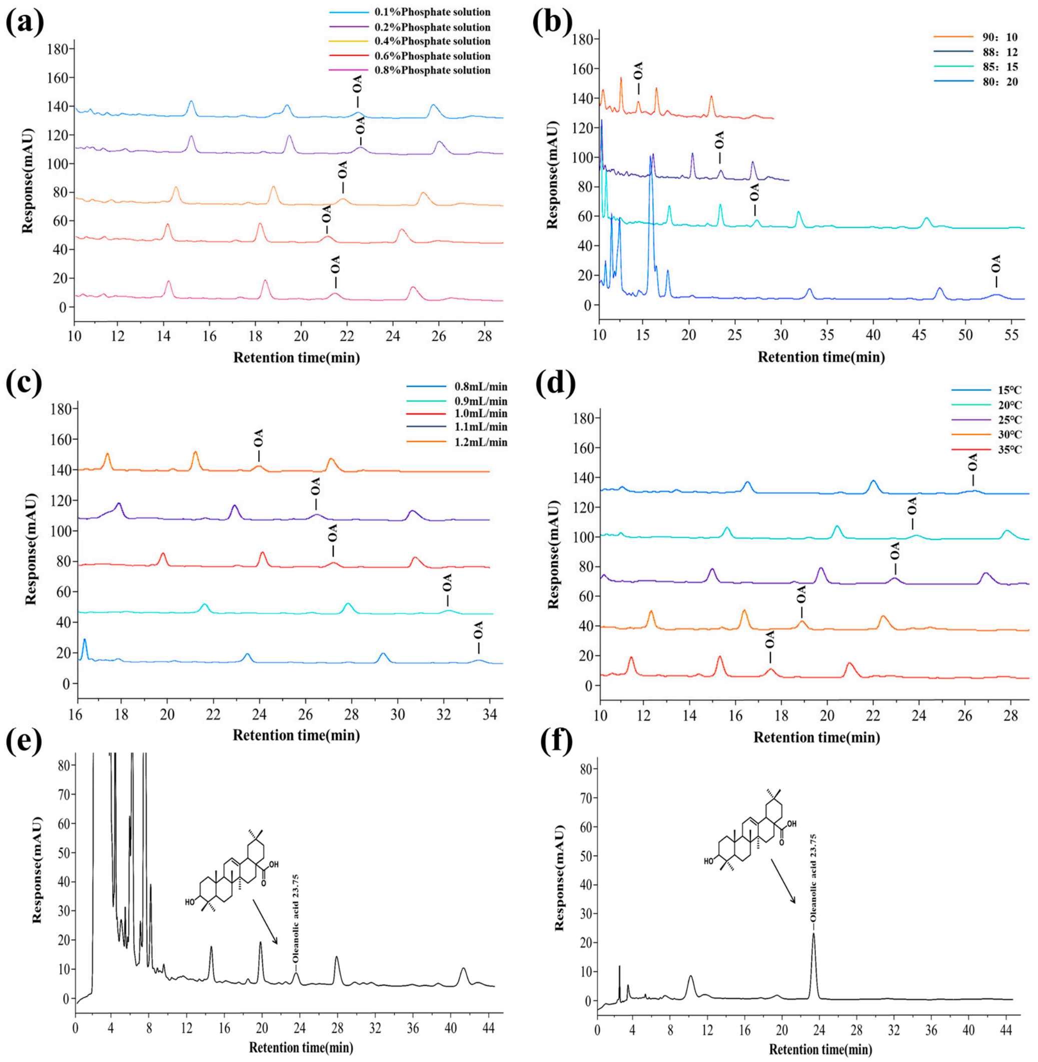

Figure 1. The effect of different chromatographic conditions on the peak time and separation of OA in BRE, as well as the optimized chromatograms of the sample and standard. (**a**) The influence of different phosphate buffer solutions; (**b**) The impact of different flow matching ratios; (**c**) The impact of different flow velocities; (**d**) The influence of different column temperatures; (**e**) Chromatogram of *Boschnikia rossica* sample; (**f**) Chromatogram of Oleanolic Acid (OA) Standard Solution.

2. This image is Figure 2 from the manuscript.

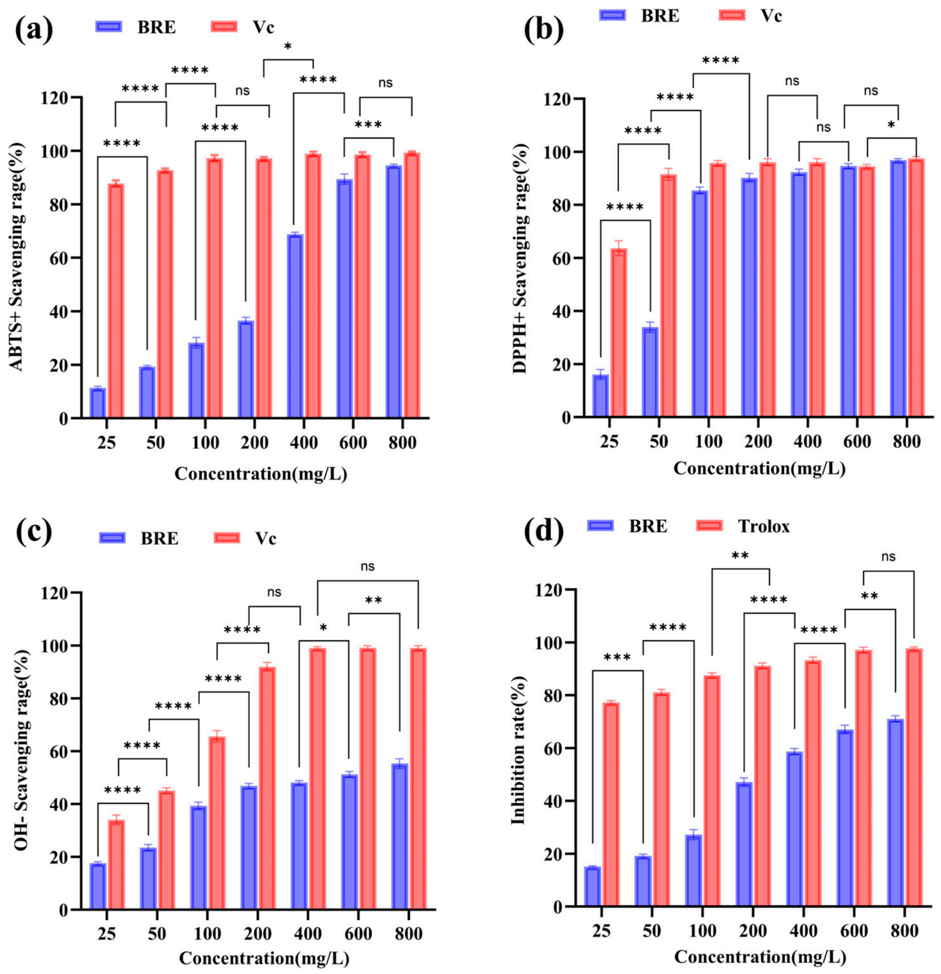

Figure 2. In vitro antioxidant activity of different concentrations of BRE. (**a**) ABTS radical scavenging ability; (**b**) DPPH radical scavenging ability; (**c**) • OH radical scavenging ability; (**d**) β-carotene bleaching inhibition ability. ****, ***, **, * and ns respectively represent $p < 0.0001$, $p < 0.001$, $p < 0.01$, $p < 0.05$, and $p > 0.05$.

3. This image is Figure 3 from the manuscript.

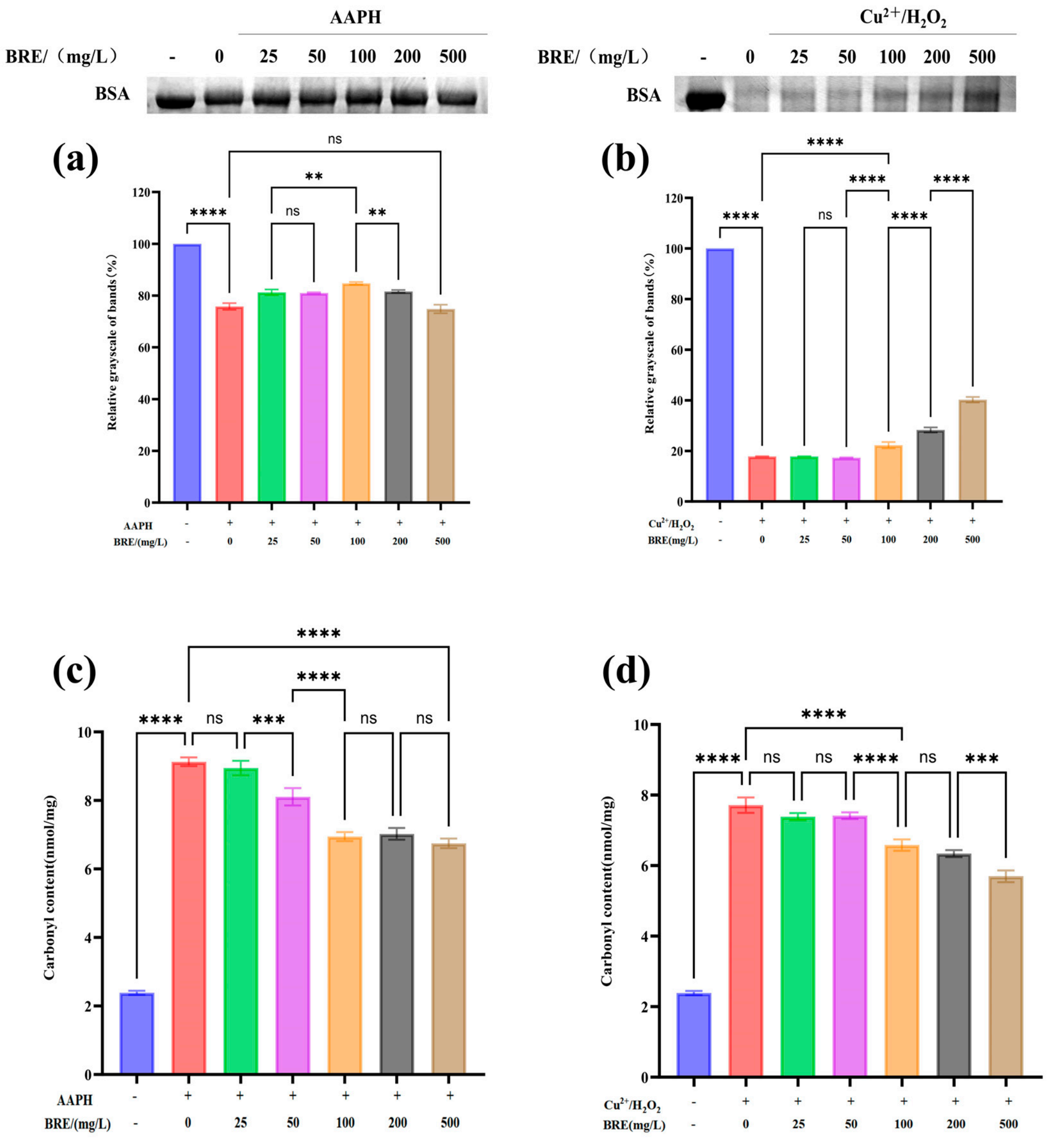

Figure 3. Protective effect of BRE on AAPH and $CuSO_4/H_2O_2$ induced BSA oxidative damage, (**a**) Protective effect of BRE on AAPH induced BSA oxidative damage; (**b**) BRE has a protective effect against $CuSO_4/H_2O_2$ induced BSA oxidative damage; (**c**) BRE induces changes in BSA carbonyl content in AAPH; (**d**) BRE induces changes in BSA carbonyl content with $CuSO_4/H_2O_2$. ****, ***, **, * and ns respectively represent $p < 0.0001$, $p < 0.001$, $p < 0.01$, $p < 0.05$, and $p > 0.05$.

4. This image is Figure 4 from the manuscript.

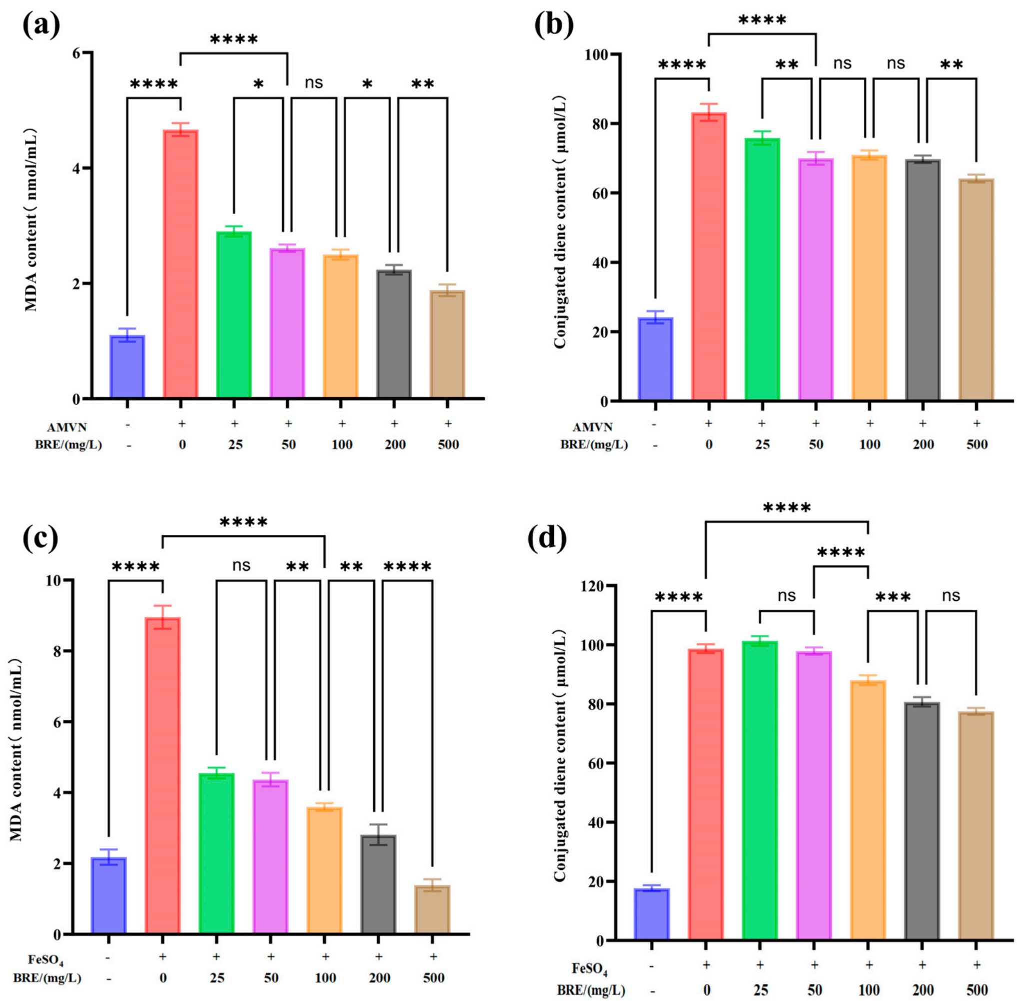

Figure 4. The effect of BRE on $FeSO_4$ and AMVN-induced LA peroxidation, (**a**) BRE induced changes in MDA content by AMVN; (**b**) BRE induces changes in conjugated diene content in AMVN; (**c**) BRE induces changes in MDA content in $FeSO_4$; (**d**) BRE induces changes in conjugated diene content induced by $FeSO_4$. ****, ***, **, * and ns respectively represent $p < 0.0001$, $p < 0.001$, $p < 0.01$, $p < 0.05$, and $p > 0.05$.

5. This image is Figure 5 from the manuscript.

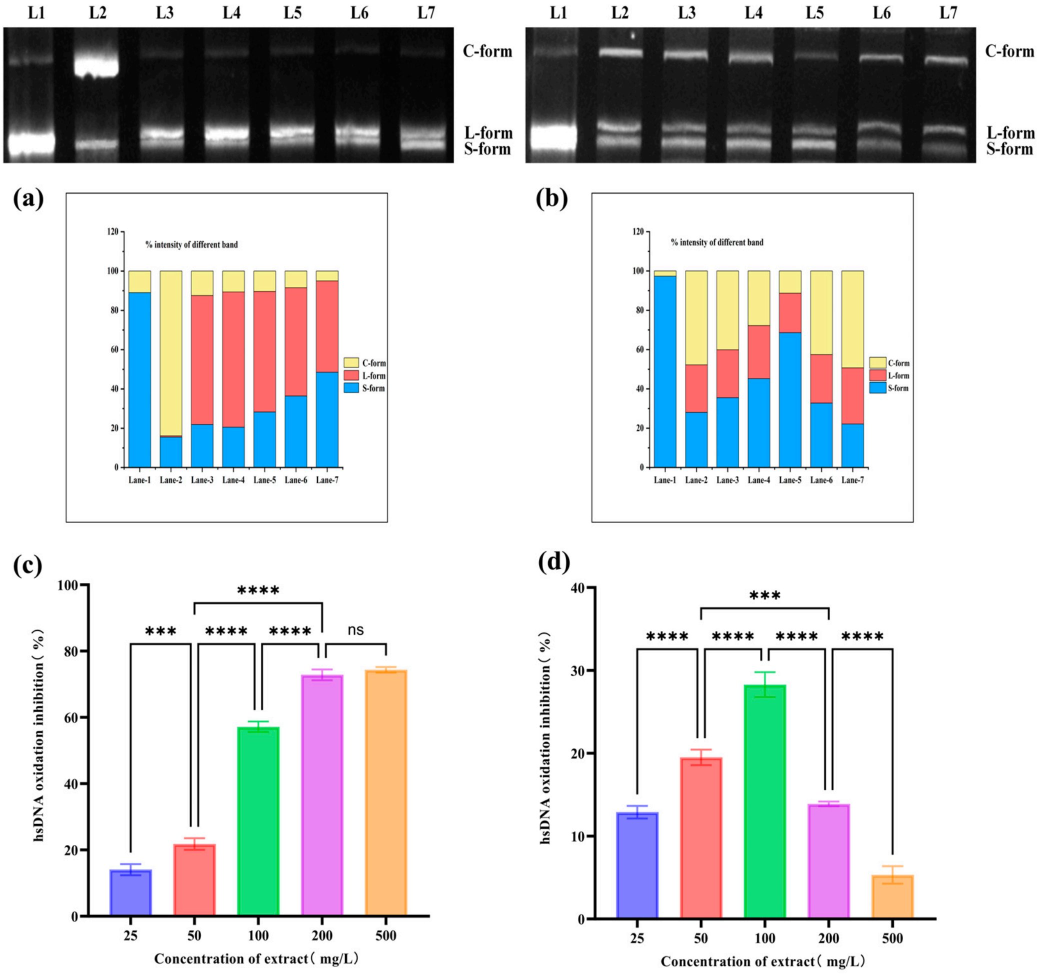

Figure 5. Effects of BRE on AAPH and $CuSO_4/H_2O_2$ induced oxidative damage to pBR322 DNA and hsDNA. (**a**) The inhibitory effect of BRE on AAPH-induced oxidation of pBR322 DNA; (**b**) The inhibitory effect of BRE on $CuSO_4/H_2O_2$ induced oxidation of pBR322 DNA. Lane-1: pBR322 DNA; Lane-2: pBR322 DNA+AAPH/$CuSO_4/H_2O_2$; Lane-3: pBR322 DNA+AAPH/ $CuSO_4/H_2O_2$+25 mg/L BRE; Lane-4: pBR322 DNA+AAPH/$CuSO_4/H_2O_2$+50 mg/L BRE; Lane-5: pBR322 DNA+AAPH/$CuSO_4/H_2O_2$+100 mg/L BRE; Lane-6: pBR322 DNA+AAPH/$CuSO_4/H_2O_2$+200mg/L BRE; Lane-7: pBR322 DNA+AAPH/$CuSO_4/H_2O_2$+500 mg/L BRE. (**c**) The inhibitory effect of BRE on AAPH-induced hsDNA oxidation; (**d**) The inhibitory effect of BRE on hsDNA oxidation induced by $CuSO_4/H_2O_2$. ****, ***, **, * and ns respectively represent $p < 0.0001$, $p < 0.001$, $p < 0.01$, $p < 0.05$, and $p > 0.05$.
